# Alteration of Cx37, Cx40, Cx43, Cx45, Panx1, and Renin Expression Patterns in Postnatal Kidneys of Dab1-/- (*yotari*) Mice

**DOI:** 10.3390/ijms22031284

**Published:** 2021-01-28

**Authors:** Mirela Lozić, Natalija Filipović, Marija Jurić, Ivona Kosović, Benjamin Benzon, Ivana Šolić, Nela Kelam, Anita Racetin, Koichiro Watanabe, Yu Katsuyama, Masaki Ogata, Mirna Saraga-Babić, Katarina Vukojević

**Affiliations:** 1 Department of Anatomy, Histology and Embryology, University of Split School of Medicine, 21000 Split, Croatia; mirelalozic3@gmail.com (M.L.); natalija.filipovic@mefst.hr (N.F.); maarjur@gmail.com (M.J.); ivona.kosovic@gmail.com (I.K.); benzon.benjamin@gmail.com (B.B.); ivana.solic1@gmail.com (I.Š.); nelakelam6@gmail.com (N.K.); anitamuic10@gmail.com (A.R.); mirna.saraga-babic@mefst.hr (M.S.-B.); 2 Department of Medical Genetics, School of Medicine, University of Mostar, 88000 Mostar, Bosnia and Herzegovina; 3 Department of Anatomy, Shiga University of Medical Science, Ötsu 520-2192, Japan; ds111799@g.shiga-med.ac.jp (K.W.); kats@belle.shiga-med.ac.jp (Y.K.); 4 Division of Anatomy, Faculty of Medicine, Tohoku Medical and Pharmaceutical University, Sendai, 981-Miyagi 8558, Japan; mogata@tohoku-mpu.ac.jp

**Keywords:** Cx37, Cx40, Cx43, Cx45, Pnx1, renin, *yotari*, kidney

## Abstract

Numerous evidence corroborates roles of gap junctions/hemichannels in proper kidney development. We analyzed how Dab1 gene functional silencing influences expression and localization of Cx37, Cx40, Cx43, Cx45, Panx1 and renin in postnatal kidneys of *yotari* mice, by using immunohistochemistry and electron microscopy. Dab1 Δ102/221 might lead to the activation of c-Src tyrosine kinase, causing the upregulation of Cx43 in the medulla of *yotari* mice. The expression of renin was more prominent in *yotari* mice (*p* < 0.001). Renin granules were unusually present inside the vascular walls of glomeruli capillaries, in proximal and distal convoluted tubules and in the medulla. Disfunction of Cx40 is likely responsible for increased atypically positioned renin cells which release renin in an uncontrolled fashion, but this doesn’t rule out simultaneous involvement of other Cxs, such as Cx45 which was significantly increased in the *yotari* cortex. The decreased Cx37 expression in *yotari* medulla might contribute to hypertension reduction provoked by high renin expression. These findings imply the relevance of Cxs/Panx1 as markers of impaired kidney function (high renin) in *yotari* mice and that they have a role in the preservation of intercellular signaling and implicate connexopathies as the cause of premature death of *yotari* mice.

## 1. Introduction

There is an increasing amount of evidence corroborating the influential roles of gap junctions and hemichannels (connexons) as factors of proper kidney development and homeostasis [1]. Connexins (Cxs), tetraspan transmembrane proteins, assemble to form gap junctions by the docking of two hemichannels of neighboring cells [2]. Each type of homo- or heterohexameric Cx-made channel has a characteristic permeability for the transfer of signaling molecules with a molecular mass of less than 1 kD. Half of all known Cxs are expressed in the human and rodent kidney and four subtypes (Cx37, Cx40, Cx43, and Cx45) are primarily localized in the gap junctions of renal vasculature which primarily contribute to renal haemodynamics [3]. For that reason, changes in normal Cx spatio- temporal- patterning are primarily observed in blood vessels. Due to their important role in intercellular communication, mutations of genes encoding some of the Cx proteins have been associated with several human diseases including blood vessel abnormalities, atherosclerosis and hypertension [4,5,6,7,8]. Cxs are not only building blocks of hemichannels, but can also aid in cell growth and cell death modulation [9,10]. Additionally, Cx knockout (KO) mice show several defects ranging from relatively mild impairments to embryonic lethality [11]. Pannexins (Panxs) form large membrane channels, that are activated by different proteases and kinases [12]. In healthy tissue, Panx1 has a role of integration of distinct stimuli into channel activation leading to ATP release [13]. Additionally, Panx1 has a possible deleterious role in paracrine signaling contributing to cell death [12,14] and diabetic nephropathy [15]. These findings implicate the relevance of Cx/Panx signaling in different organ systems of the body [7]. Data concerning their essential role in normal cell functioning and their relation to the development of kidney diseases is still scarce. Additionally, there apparently seems to be a great deal of discrepancies concerning the specificity of expression of separate Cx isoforms in distinct regions and structures of the kidney. Unfortunately, immunohistochemistry experiments have generated conflicting data concerning Cx expression and localization due to limitations of commonly used Cx antibodies and the sensitivity of experimental settings. Because of this, one of the goals of our research is to help answer the question of Cxs expression outside the vascular wall.

Signaling via Cxs seems to have a major role in normal kidney development, during the postnatal period as well as in kidney detrimental effects [16,17]. In renal blood vessels, Cx37, Cx40, Cx43, and Cx45 are expressed in the layers of the vessel wall, with the most prominent expression of Cx40 in the endothelial cells and Cx45 in the smooth muscle cells blood vessels [1]. In the tubules, there is morphological evidence of the presence of gap junction plaques only in the proximal tubules [1], while the expression of other Cxs characterizes different parts of nephron tubules both during normal human development and in the postnatal period [17]. Among different Cxs, Cx40-/- mice had hypertension associated with high plasma renin activity [18,19]. Cx43 and Panx1 have been shown to be expressed in the kidneys and involved in the control of the process leading to tissue inflammation [20]. Additionally, Cx43 diminished renal fibrosis through c-Src [21] and reduced the progression of chronic kidney disease (CKD) in mice [22]. The blocking of Cx43 mediated hemichannel activity protected against early tubular injury in experimental CKD [23]. Additionally, Cx43 was involved in the progression of acute kidney injury by regulating intracellular oxidative status [24]. Research on Cx37-/- mice provided evidence that Cx37 selectively influences Ang II signaling, and accordingly reduces hypertension via modulation of the expression of the Ang II type 2 receptor [5]. Similarly, in the unilateral renal artery stenosis mice model, Cx45 prevents hyperreninemia and decreases hypertension [19].

Yotari mice, obtained by a spontaneous mutation in the Dab1 gene, exhibit histological abnormalities in the central nervous system [25,26], very similar to those of reeler (Reelin-/-) mice, suggesting that Reelin and Dab1 belong to the same signaling pathway [25,26,27]. These mice die by the end of the 3rd postnatal week, but the reason is still unknown. Recently, we found expression of DAB1 and REELIN in different kidney structures during normal human development and in postnatal human kidneys, implying their possible regulatory role in tubular formation or functional maintenance [28]. Our unpublished data implicates chronic kidney disease (CKD) as the reason for the premature death of Yotari mice, which can be propagated by a variety of mechanisms that may affect the kidney structures including blood vessels, glomeruli, and the tubulointerstitial section [29].

In light of this new information, this study aimed to analyze how Dab1 gene functional silencing influences expression and localization of Cx37, Cx40, Cx43, Cx45, Panx1, and renin in the postnatal kidneys. Namely, expression of those Cxs, Pnx1, and renin may have a crucial role in the preservation of intercellular signaling within healthy kidney tissue as well as in the prevention of CKD.

## 2. Results

We analyzed the immunoexpression of Cx37, Cx40, Cx43, Cx45, Panx1 and renin in *yotari* (Dab1-/-) and wild type postnatal mouse kidneys differing between the cortex and medulla, with a special focus on co-localization with α-Smooth muscle actin (aSMA), an actin isoform that predominates in smooth muscle cells that line the intramuscular blood vessel walls.

### 2.1. Cx37 Expression

**Figure 1:**
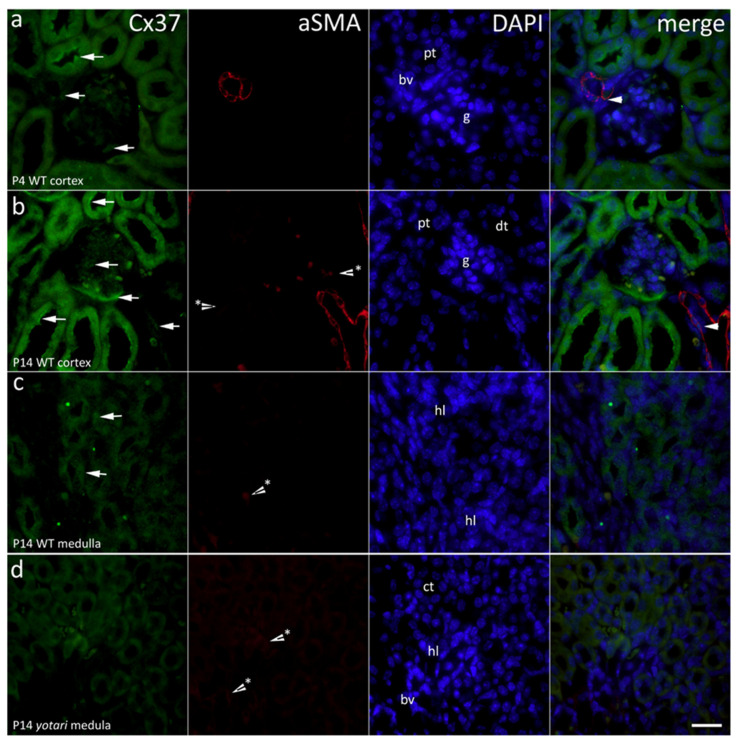
Immunofluorescence staining of postnatal *yotari* (**d**) and wild type (**a**,**b**,**c**) mouse kidneys with the Cx37 marker and co-expression of Cx37 and aSMA with DAPI nuclear staining. Expression of Cx37 (arrows), collecting tubules (ct), blood vessels (bv), glomeruli (g), loop of henle (hl), proximal convoluted (pt), and distal convoluted tubules (dt). Co-expression of Cx37 and aSMA (arrowheads) can be seen on merged photographs. Autofluorescence of nephron tubules and erythrocytes (*), not to be mistaken with positive immunofluorescence staining. Scale bar is 20 μm, refers to all images.

In the cortex of wild type mice kidneys (4th postnatal day), moderate expression of Cx37 was observed in the apical cytoplasm of proximal and distal tubules and Bowman’s capsule and very mildly in the walls of blood vessels. Moderate A- SMA staining characterized the smooth muscle cells in the vascular wall. Cx37 and A-SMA staining co-localized in the walls of blood vessels (Figure 1a). In the 14th postnatal day, cortical expression of Cx37 increased strongly in both proximal and distal tubules, in parietal epithelial cells of glomeruli, and in the wall of nearby blood vessels.

**Figure 2:**
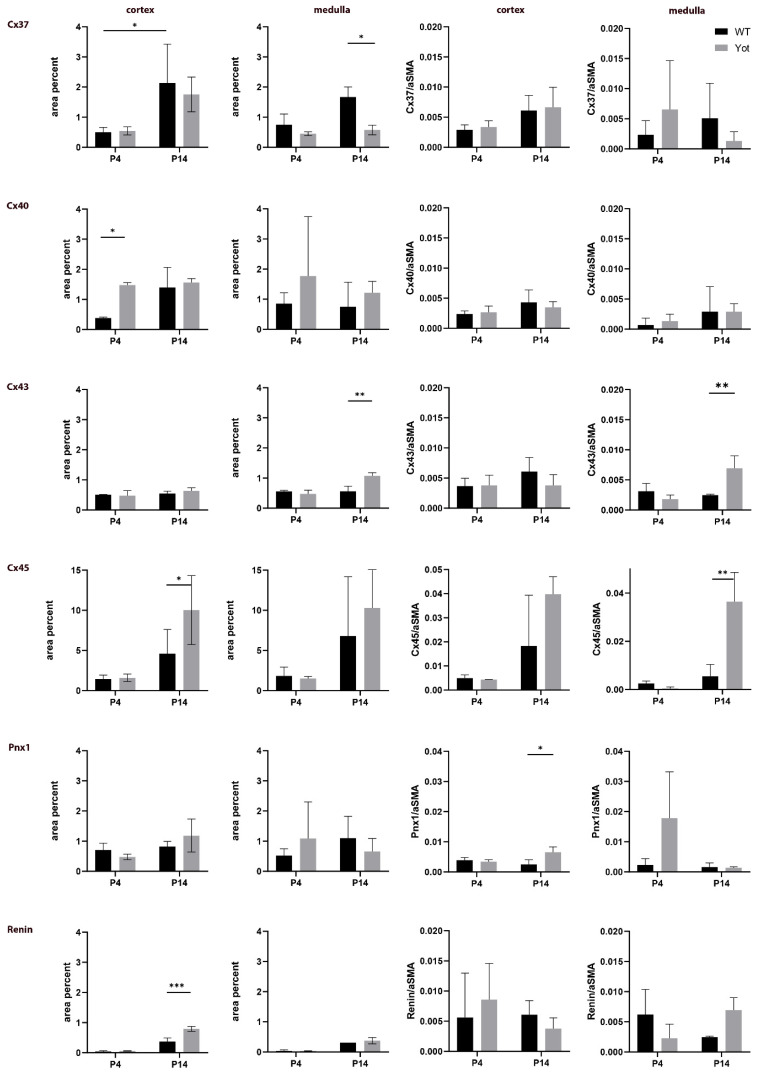
The area percentages of Cx37, Cx40, Cx43, Cx45, Panx1 and renin in the cortex and medulla of wild type and *yotari* mouse kidneys and their co-localization with α-Smooth muscle actin (aSMA). Data is presented as the mean ± SEM (vertical line). Significant differences were indicated by * *p* < 0.05, ** *p* < 0.001, *** *p* < 0.0001 (Two-way ANOVA followed by LSD multiple comparison test).

Co-localization of Cx37 and A-SMA expression was observed in the walls of blood vessels (Figure 2). Analyses revealed significant differences between the two control groups. Specifically, the expression was far more vast in 14P wild type mouse kidney cortex than in 4P (*p* < 0.05; Figure 2).

In the medulla, mild punctate expression of Cx37 was seen in the parts of the loop of Henle, while very mild expression to A-SMA was observed in thin-walled medullary blood vessels (Figure 2). In the 14th postnatal day of *yotari* mice kidneys, an approximately threefold decrease in expression of Cx37 was seen in the medulla (Figure 2) (*p* < 0.05; Figure 2). Generally, Cx37 was more present in 14P mice, both in the cortex and the medulla of the kidney, regardless of genotype (Figure 2). Semi-quantitative analysis revealed mild staining intensity in the glomeruli of all groups except 14P WT where they were moderately intense. Tubules displayed moderate staining in WT 4P and strong in 14P, while in *yotari* proximal convoluted tubules (PCTs) and distal convoluted tubules (DCTs) differed. In 4P, PCTs stained moderately and DCTs strongly, while in 14P PCTs were mild and DCTs moderate. The medulla was mildly reactive, except for 4P *yotari* which had a strong signal in the medulla (Table 1).

### 2.2. Cx40 Expression

**Figure 3:**
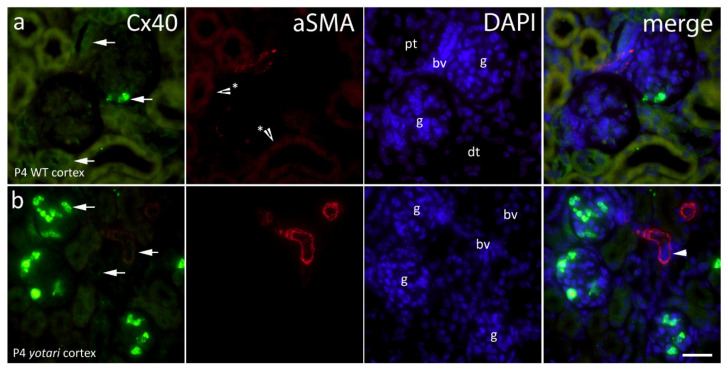
Immunofluorescence staining of postnatal *yotari* (**b**) and wild type (**a**) mouse kidneys with the Cx40 marker and co-expression of Cx40 and aSMA with DAPI nuclear staining. Expression of Cx40 (arrows), collecting tubules (ct), blood vessels (bv), glomeruli (g), proximal convoluted (pt), and distal convoluted tubules (dt). Co-expression of Cx40 and aSMA (arrowheads) can be seen on merged photographs. Autofluorescence of nephron tubules and erythrocytes (*), not to be mistaken with positive immunofluorescence staining. Scale bar is 20 μm, refers to all images.

**Figure 4:**
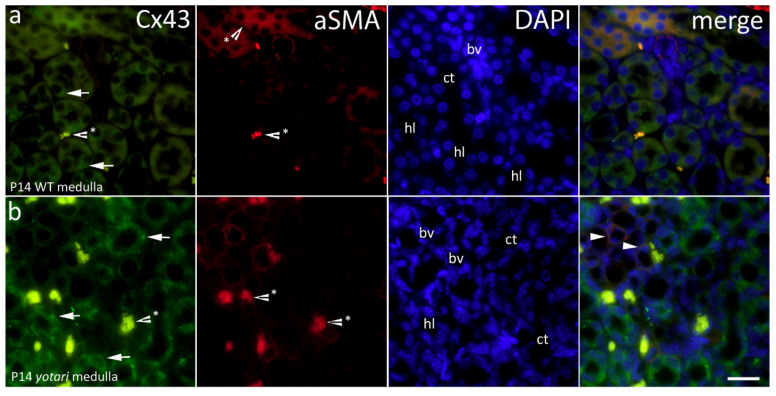
Immunofluorescence staining of postnatal *yotari* (**b**) and wild type (**a**) mouse kidneys with the Cx43 marker and co-expression of Cx43 and aSMA with DAPI nuclear staining. Expression of Cx43 (arrows), collecting tubules (ct), blood vessels (bv), loop of henle (hl) and distal convoluted tubules (dt). Co-expression of Cx43 and aSMA (arrowheads) can be seen on merged photographs. Autofluorescence of nephron tubules and erythrocytes (*), not to be mistaken with positive immunofluorescence staining. Scale bar is 20 μm, refers to all images.

In the analyses of Cx40, both punctate and diffuse cytoplasmatic staining was detected in nephron tubules and glomeruli. In the 4th postnatal day, Cx40 is strongly expressed in the glomeruli of certain cell types in wild type kidneys (Figure 3a), while in the *yotari* mice of the same age Cx40 increases in the glomerular cell population (Figure 3b). These cells were not to be mistaken with erythrocytes, which emit a red signal (autofluorescence) that penetrates onto the green filter showing yellow staining (Figure 4a,b) since the signal remained after the subtraction of the red signal using ImageJ. Results have shown a significant increase in the immunoexpression of Cx40 in the 4P *yotari* mouse kidney cortex in comparison to control animals (*p* < 0.05; Figure 2). The expression does not vary much in 14P wt and yot in comparison to 4P yot. As for the semi-quantitative analysis, WT mice at day 4P showed mild staining intensity in the glomeruli and medulla, and moderate in the tubules, while 14P displayed moderate reactivity in the DCTs and mild in the rest of the structures. Regardless of age, *yotari* tubules in the cortex and medulla were mildly to moderately positive, but the glomeruli were stronger in intensity in the younger (4P) kidneys (Table 1).

### 2.3. Cx43 Expression

We observed difuse staining of Cx43 that did not discriminate between different structures of the kidney. In the cortex, fluorescence appeared steady regardless of postnatal day and genotype. On the other hand, a significant increase of Cx43 immunoexpression in the medulla of *yotari* mouse kidneys was found on 14P compared to the control group (*p* < 0.01; Figure 2). In the 14th day of wild type mice, parts of the medulla such as the thick and thin segment of the loop of Henle show mild cytoplasmic expression of Cx43 (Figure 4a), while on the 14th day of the *yotari* medulla, slightly stronger expression of Cx43 compared to the wild type is observed in thick and thin segments of the Loop of Henley (Figure 4b). Walls of blood vessels show immuno reactivity to A-SMA (Figure 4). In addition, a statistically significant difference was found between the co-localization of Cx43 with aSMA (calculated by dividing the area of overlap with aSMA area) in the medulla, with there being much more spatial overlap in *yotari* samples (*p* < 0.01; Figure 2). Concerning intensity of immunoreactivity, 4P mice were mild in all structures, regardless of genotype, aside from *yotari* glomeruli which were moderate. 14P WT showed mild reactivity in the glomeruli, PCTs and the medulla, and moderate in the DCTs. Yotari mice were similar, except for the already mentioned stronger expression in the medulla (Table 1).

### 2.4. Cx45 Expression

**Figure 5:**
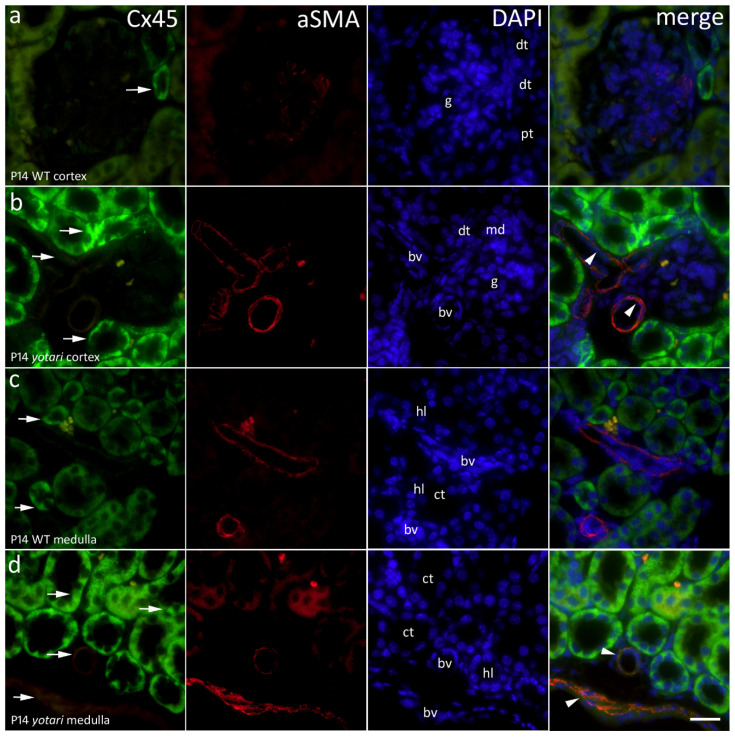
Immunofluorescence staining of postnatal *yotari* (**b**,**d**) and wild type (**a**,**c**) mouse kidneys with the Cx45 marker and co-expression of Cx45 and aSMA with DAPI nuclear staining. Expression of Cx45 (arrows), collecting tubules (ct), blood vessels (bv), macula densa (md), glomeruli (g), loop of henle (hl), proximal convoluted (pt), and distal convoluted tubules (dt). Co-expression of Cx45 and aSMA (arrowheads) can be seen on merged photographs. Scale bar is 20 μm, refers to all images.

An abundance of strong and diffuse cytoplasmatic staining was seen in tubules and some punctate fluorescence only sporadically in renal corpuscles (Figure 5). However, there was an extensive amount of strong Cx45 immunoreactivity displayed in 14P *yotari* mice, both in the cortex and in the medulla (Figure 5b,d). Compared to that group, 14P wt mice had significantly less of the Cx45 protein in the cortex, mostly some moderate expression in the distal tubules (*p* < 0.05; Figure 2 and Figure 5a), while in the medulla, the thin descending parts of the loop of Henle displayed moderate immunoreactivity. Additionally, a statistically significant difference was found concerning the co-localization of Cx45 with aSMA in the blood vessels of the medulla, with there being much more spatial overlap in *yotari* samples (*p* < 0.01; Figure 2 and Figure 5c,d). Similarly, there was far less expression in 4P mice, regardless of group. Immunoreactivity was mild in the glomeruli and PCTs, and moderate in DCTs and the medulla of 4P WT mice. Same age *yotari* specimens displayed stronger intensity in every structure type except for the glomeruli where it remained mild. Cortical tubules of WT and *yotari* mice age 14P were strong, the glomeruli and medulla of WT were moderate, *yotari* glomeruli were only mild while the medulla was strong (Table 1).

### 2.5. Panx1 Expression

**Figure 6:**
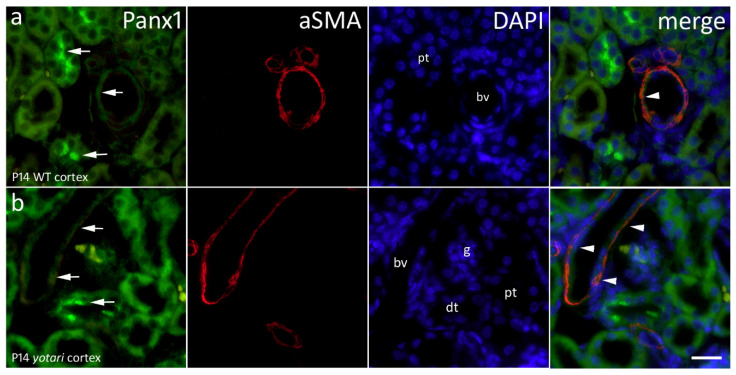
Immunofluorescence staining of postnatal *yotari (***b***)* and wild type (**a**) mouse kidneys with the Panx1 marker and co-expression of Panx1 and aSMA with DAPI nuclear staining. Expression of Panx1 (arrows), blood vessels (bv), glomeruli (g), proximal convoluted (pt) and distal convoluted tubules (dt). Co-expression of Panx1 and aSMA (arrowheads) can be seen on merged photographs. Scale bar is 20 μm, refers to all images.

In the control group, Panx1 is moderately expressed in the apical cytoplasm of proximal and distal tubules and the cytoplasm of cells within the glomeruli and mildly in blood vessels of wild type kidneys in the 14th postnatal day (Figure 6a). In 14th day *yotari* kidneys, expression of Panx1 increases in both proximal and distal tubules, while its expression in blood vessels is still very mild. The aSMA, which characterizes blood vessels, co-expresses with Panx1 in the walls of blood vessels. Statistically, the only significant result found was the difference in area percentage of co-localization between Panx1 and aSMA in the cortex. The cortex of 14P *yotari* mice contained much more spatial overlap than controls of the same postnatal day (*p* < 0.05; Figure 2). Semi-quantitative analysis revealed mild staining intensity in the glomeruli and PCTs in WT and *yotari* 4P. Additionally, both genotypes at that age lacked reactivity in the medulla, while the older group displayed mild intensity staining. DCTs were moderate in WT and strong in *yotari*, regardless of age. Lastly, the glomeruli and PCTs of 14P WT were moderately stained, while in *yotari* the glomeruli were noticeably stronger in intensity (Table 1).

### 2.6. Renin Expression

**Figure 7:**
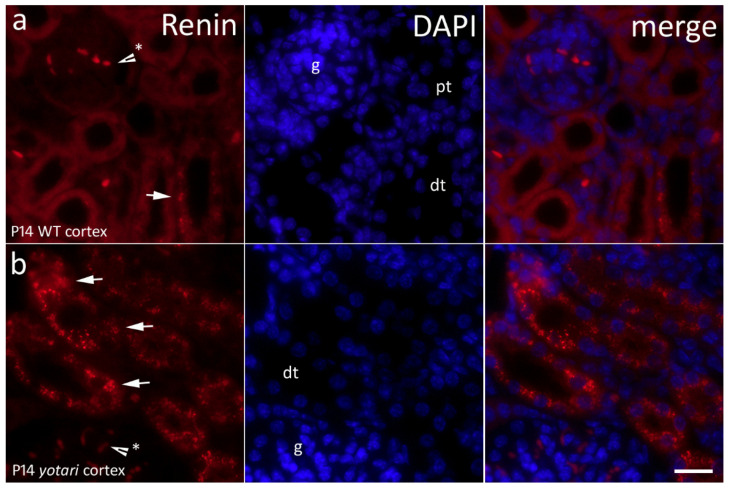
Immunofluorescence staining of postnatal *yotari* (**b**) and wild type (**a**) mouse kidneys with the renin marker and DAPI nuclear staining. Expression of renin (arrows), glomeruli (g), proximal convoluted (pt), and distal convoluted tubules (dt). Autofluorescence of nephron tubules and erythrocytes (*), not to be mistaken with positive immunofluorescence staining. Scale bar is 20 μm, refers to all images.

**Figure 8:**
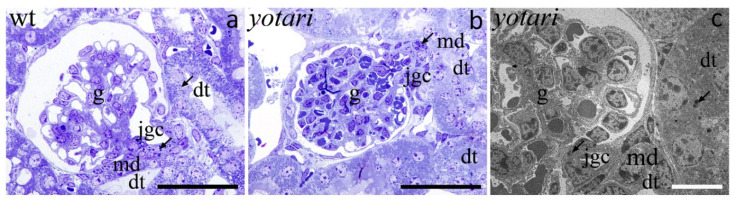
Light microscopy (LM) and transmission electron microscopy (TEM) images of wild type and *yotari* kidneys on P14. Semi-thin sections of the kidneys of wild type animals with typical histological structure (**a**). Semi-thin sections of the kidneys of *yotari* animals with juxtaglomerular apparatus and renin granules in different kidney structures (arrows) (**b**). Representative electron microscope photograph of glomeruli with juxtaglomerular apparatus (**c**). Renin granules (arrows), glomeruli (g), macula densa (md), juxtaglomerular apparatus (jgc), and distal convoluted tubules (dt). Scale bars are 50 μm (**a**,**b**) and 10 μm (**c**).

Grains of renin gave off an intense red signal in distal convoluted tubules of the kidney cortex and luminal staining of a portion of the tubules (Figure 7). The expression of renin in the 4P cortex was minimal compared to 14P. Furthermore, in 14P specimens, we observed a particularly relevant find of renin being much more prominent in *yotari* mice than in controls (*p* < 0.001; Figure 2). On our light microscopy (LM) and transmission electron microscopy (TEM) images of wild type and *yotari* kidneys on P14, we can see renin granules in the juxtaglomerular apparatus and in distal convoluted tubules (Figure 8).

## 3. Discussion

Seeing as Cxs are expressed in all parts of the nephron and in the renal vasculature, it is justifiable to speculate about their role in some kidney pathologies, with dysfunction of connexin proteins as the basis of, or at least a contributor to CKD [16,30]. Using a model of Dab1-/- mice we characterized the expression pattern of several Cxs and Panx1 as key molecular agents implicated in renin cell activation in those mice.

**Figure 9:**
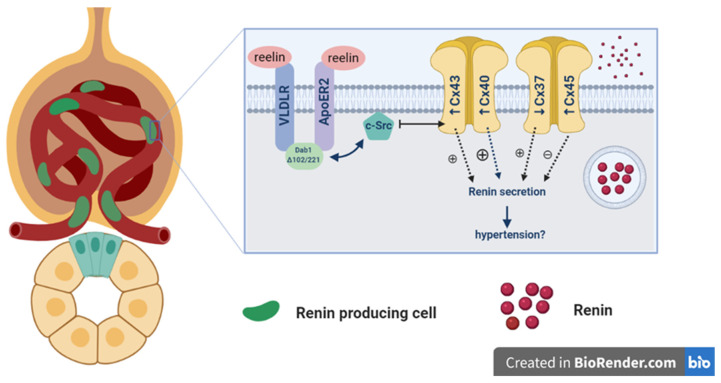
Yotari mice, obtained by a spontaneous mutation in the Dab1 gene, produce Dab1 Δ102/221 which might lead to the activation of c-Src tyrosine kinase, causing the upregulation of Cx43 in the medulla of the kidney. This overexpression leads to renin secretion. Atypically positioned renin producing cells which release renin in an uncontrolled fashion are most likely the result of disfunction of Cx40, but this does not rule out simultaneous involvement of other Cxs, such as Cx45 which was significantly increased in the *yotari* cortex. Cx45 prevents hyperreninemia and decreases hypertension. The physiological role of Cx37 would be to inhibit renin secretion and in consequence to lower blood pressure, thus a decreased expression in the *yotari* medulla might contribute to hypertension. Alteration of Cx expression in *yotari* mice could be an early signal for the development of chronic kidney disease (CKD).

Given the involvement of different Cxs in the control of numerous cellular processes, our findings might have some importance regarding the prevention of CKD. Namely, we propose that alteration of Cx expression in *yotari* mice is an early signal for the development of CKD. This assumption is in accordance with the study of Toubas et al. on a transgenic strain of hypertensive RenTg mice, which also suggested the importance of Cxs as markers of chronic renal disease by their participation in the inflammatory process during the development of kidney pathology [31]. Thus, our results point out the importance of Cxs in cell responses to silenced Dab1 in *yotari* mice and their possible connection to the Dab1/Reelin signaling pathway (Figure 9). In our study, the expression of Cxs in the kidney tissue of wt mice was predominantly very low in comparison to *yotari*, except for Cx37 that was markedly increased, especially in the medulla of wt mice at P14. In contrast, Cx40 expression was significantly higher in the cortex of P4 *yotari*, followed by higher expression of Cx45 in the cortex and Cx43 in the medulla of P14 *yotari*.

Previous animal studies have demonstrated expression of Cx37 primarily in the thick ascending limb of Henle and the distal convoluted tubule, but less so in the proximal tubule and collecting duct [32]. In our study, similar results were obtained: in the cortex of wild type mice kidneys, moderate expression of Cx37 was observed in the apical cytoplasm of proximal and distal tubules and Bowman’s capsule and very mildly in the walls of blood vessels, while in the medulla, mild punctate expression of Cx37 was seen in the parts of the loop of Henle. Research on Cx37 null mice showed that Cx37 may counter the effect of Cx43 on atherosclerosis [5]. In our study, in the *yotari* cortex at P14, Cx37 expression was lower in regard to Cx43. In a study by Jose et al., an increase in the Cx43/Cx37 ratio was shown to be a regulator of renal fibrosis [33]. Additionally, propagation of vasodilatation was decreased in Cx40 knockout mice, while in Cx37 knockout mice propagation of vasoconstriction was observed [1]. In our study, Cx37 and Cx40 displayed inverse appearance, with higher expression of Cx37 in comparison to Cx40 (in wt), while in *yotari* mice the expression of Cx40 was always higher than in wt, thus implying a regulatory role of Cxs in overcoming high renin expression in *yotari*. In addition, we also observed decreased Cx37 expression in the P14 *yotari* medulla that might contribute to hypertension reduction provoked by high renin expression. Namely, Cx37-/- mice reduce hypertension via modulation of the expression of the Ang II type 2 receptor [5].

Activation of cAMP pathways was shown to be connected to renin secretion. Namely, renin, as a main modulator of the renin-angiotensin system, is an important link in the regulation of extracellular fluid volume and blood pressure control [34]. Unpublished results seem to show less extracellular fluid volume in *yotari* kidneys, but for further confirmation, blood pressure measurements are needed. Renal juxtaglomerular (JG) cells, which are mainly responsible for renin secretion, are controlled by several factors with different underlying signaling pathways but with the common ultimate aim of changing the concentration of the intracellular Ca^2+^ [35,36]. Low Ca^2+^ in JG cells increases intracellular cAMP which stimulates renin secretion [35,37,38]. One of the interesting finds in our study is that renin granules were observed not only in the juxtaglomerular apparatus (JGA) but also inside the vascular walls of the glomeruli capillaries, in the proximal and distal convoluted tubuli as well as in the medulla resembling the distribution in early embryonic and fetal life [34]. It is known that cells derived from the renin line are capable of dedifferentiation in the case of a homeostasis crisis [34].

This provides a broader network of activity and intercellular communication concerning Cx control of renin secretion. Recently, Hong and Yao proposed that using strategies such as specific siRNA or Cxs mimetic peptides might lead to the development of more effective approaches in the control of renin secretion in vivo [39]. In the last decade, the role of gap junctions in the control of renin has been extensively investigated and multiple lines of evidence have shown that Cx40 channels are required for regulatory mechanisms of renin secretion and associated with correct localization of cells that express renin [19]. Thus, increased renin secretion and associated hypertension have been observed in Cx40 knockout mice and mice with genetically engineered Cx45 reduction in JG cells [40,41]. Using the mouse renin-secreting cell line, Hong and Yao observed that activation of Cx hemichannels is an important step in low Ca^2+^ induced activation of the cAMP pathway, and therefore induction of renin secretion. Additionally, blockade of Cx hemichannels or downregulation of Cxs in renin-secreting cells caused the termination of cAMP pathway activation [39]. In our study, we found high Cx40 expression in the cortex of *yotari* mice in P4 and it was significantly higher in regard to wt, while consequently renin expression dramatically increased in the cortex of P14 *yotari* mice. This finding is in accordance with the previous report that disturbance of Cx40 intercellular signaling may lead to a disbalance of blood pressure control through renin secretion and hypertension onset [39]. Genetic loss-of-function defects of Cx40 in renal JG cells have been associated with renin-dependent hypertension [42]. Similarly, we found a significant increase of Cx40 in the cortex of P4 *yotari* mice, which corresponded to normal renin expression. However, when this regulatory mechanism was exhausted, even normal expression of Cx40 at P14 *yotari* mice was not enough to prevent a high increase of renin expression at P14. Our conjecture is that the brightly fluorescing cells found in the glomeruli of P14 *yotari* mice are renin producing cells because Cx40 is known to be their most abundant connexin which displays strong expression [34]. We also believe that the hypersecretion of renin is caused by some kind of disfunction of Cx40 in the renin cells due to the fact that Cx40 deficient mice had a high number of atypically positioned renin cells which released renin in an uncontrolled fashion [43,44]. Additionally, the Cx40 dependent effects do not rule out the possible simultaneous involvement of other Cxs, such as Cx45 which was significantly increased in the cortex of P14 *yotari* mice, and was paralleled by an increase in renin expression. This result is in accordance with the findings of Li et al., who discovered that the hypertension-induced elevation of Cx45 may influence communication between vascular smooth muscle cells and endothelium, resulting in an increased vasoconstrictive response and development of hypertension [6]. The substitution of Cx40 with Cx45 was shown to prevent hyperreninemia and decreased hypertension in the unilateral renal artery stenosis mice model [19]. Interestingly, mice in which Cx40 was replaced by Cx45 had weaker steady-state autoregulation and tubuloglomerular feedback than wild-type mice, but stronger than Cx40 KO mice, suggesting that Cx45 can partially mimic Cx40 functions [45].

Our result of higher expression of Cx43 in the medulla of P14 *yotari* mice might be in line with the increased Cx43 expression in nephropathy, where Cx43-mediated ATP release represented an initial trigger of early tubular injury via its actions on the adherents and tight junction complex. Thus, Cx43 might represent a novel target for intervention on tubulointerstitial fibrosis in CKD [23]. However, there is contradicting information on the function of Cx43 in kidney pathology: while Cx43 upregulation in the glomeruli was described in experimental rat glomerulonephritis and type 2 diabetes, its downregulation was observed in overt diabetic nephropathy). One of the downstream targets of Dab1 is c-Src tyrosine kinase through which Cx43 was shown to diminish renal fibrosis [21]. Accordingly, we suggest that loss of Dab1 might lead to the activation of c-Src tyrosine kinase, resulting in the upregulation of Cx43 and subsequent attenuation of renal fibrosis and electric abnormalities by activation of the cyclic adenosine monophosphate (cAMP) pathway. The increased expression of Cx43 in *yotari* mice that we found in our study is in support of our hypothesis. However, this link by which Cxs cross the Dab1/Reelin pathway remains to be elucidated.

Panx1 has been shown to be involved in ATP release, as Panx1 channels open and release ATP when the intracellular Ca^2+^ level is increased [46]. In our study, Panx1 expression was higher in the P14 *yotari* cortex than in the control, but without statistical significance, therefore further research is needed in elaborating the role of Panx1 kidney pathology.

The expression of Cxs in renal blood vessels is proposed to have a great impact on the regulation of renal blood flow, arteriolar tonus and blood pressure control [47,48]. In our study, Cx37 and Cx40 expression pattern was paralleled closely by that of the vascular marker aSMA. The high expression of Cx43 and Cx45 in the vasculature of P14 *yotari* mice compared to control mice might imply the increased magnitude of conducted vasomotor response. Studies suggest that high blood pressure induces changes in the expression of vascular connexins in a region dependent fashion implying secondary changes in vascular function, thus implying possible hypertension could be the cause of these statistically relevant findings [49]. Contrary to our results, Braunstein et al. found an abundance of Cx37 in arterioles from normotensive rats compared to hypertensive ones [50]. For a better evaluation in future studies, we would need to primarily assess the renal function, among other assessments, of the two groups of mice to observe any differences in parameters such as blood pressure, which is a shortcoming of our current study.

It is reasonable to expect comorbidities in the other organs that express the same connexin subtypes as the kidney if Cx spatio-temporal disturbances are found. However, since connexins are expressed in diverse quantities and different temporal patterns, their dysfunction might not be obvious or certain compensatory mechanisms could rescue the phenotype [4]. Additionally, different combinations of connexin subtypes in organs could explain this enigma. However, possibly the simplest answer could be that the restricted presence of Dab1 could be responsible for the existence of kidney and neuronal pathologies in *yotari* mice and the absence of other obvious connexopathies.

## 4. Materials and Methods

### 4.1. Generation of Dab1 Conventional Mutants and Sample Collection

The experiment was performed using C57BL/6N mice which were group-housed in regulation polycarbonate cages with free access to food and tap water, in a temperature-controlled (23 ± 2 °C) room with a 12-h light/dark cycle. Yotari (Dab1-/-) mice, constructed as previously described [25,27,30], were used as Dab1 null conventional mutants. For genotyping, we used the following PCR primers: *yotari*—GCCCTTCAG-CATCACCATGCT and CAGTGAGTACATATTGTGTGAGTTCC, wild type—GCCCTTCAGCATCACCATGCT and CCTTGTTTCTTTGCTTTAAGGCTGT [31]. The mice were sacrificed on either their 4th or 14th postnatal day (4P, 14P). First, they were anesthetized with pentobarbital and afterward transcardially perfused using phosphate buffer saline (PBS, pH 7.2) and 4% paraformaldehyde (PFA) in 0.1 M PBS. Their kidneys were separately fixed with 4% PFA in 0.1 M PBS overnight for Hematoxylin-Eosin (HE) and immunofluorescence staining and with a 2% PFA + 2.5% glutaraldehyde (GA) mixture for light microscopic and ultrastructural studies.

### 4.2. Immunofluorescence

After fixation, tissue was dehydrated with graded ethanol solutions, embedded in paraffin blocks and serially cut as 5 µm-thick sections which were then mounted on glass slides. Proper tissue preservation was confirmed by HE staining of every 10th section. Following deparaffinization in xylol and rehydration in graded ethanol and distilled water, the mounted tissue samples were heated in a sodium citrate buffer for 20 min at 95 °C in a water steamer and then gradually cooled down to room temperature. Protein blocking buffer (ab64226, Abcam, Cambridge, UK) was then applied for 30 min to prevent non-specific staining. The samples were then incubated with primary antibodies (Table 2) overnight in a humidity chamber. The following day, they were rinsed with PBS before being incubated with suitable secondary antibodies (Table 2) for one hour. Finally, the samples were washed in PBS once more, nuclei were stained blue using 40,6-diamidino-2-phenylindole (DAPI) and then samples were cover-slipped (Immuno-Mount, Thermo Shandon, Pittsburgh, PA, USA). No staining was observed when primary antibodies were omitted from the immunofluorescence protocol.

### 4.3. Tissue Preparation for Light Microscopy (LM) and Transmission Electron Microscopy (TEM)

After fixation, the tissue samples were washed with PBS and post-fixed in 2% osmium tetroxide for 2 h. Succeeding dehydration in graded ethanol, samples were embedded in TAAB Epon 812 (TAAB, Reading, UK). The tissue samples were cut into semi-thin sections (300 nm in thickness) with an Ultracut UCT ultramicrotome (Leica Microsystems, Wetzlar, Germany) equipped with a glass knife. The semi-thin sections were mounted on glass slides, stained with toluidine blue and examined by LM. Ultra-thin sections (70 nm in thickness) were cut on the same microtome equipped with a diamond knife. The ultra-thin sections were stained with 1% uranyl acetate and lead citrate and examined on a JEM 1400 transmission electron microscope (JEOL, Tokyo, Japan) operated at 80 kV.

### 4.4. Data Acquisition and Statistical Analysis

While being examined by a fluorescence microscope (Olympus BX51, Tokyo, Japan) equipped with a Nikon DS-Ri1 camera (Nikon Corporation, Tokyo, Japan), images of mouse kidney cortex and medulla were taken at 40× magnification to be used for analysis. Images used for the assembling of most figures were taken at 100× magnification. For electron microscopy, a JEM 1400 TEM (JEOL, Tokyo, Japan) operated at 80 kV and set up with a JEOL charge-coupled device (CCD) camera system (Advanced Microscopy Techniques, Danvers, MA, USA) was used.

**Figure 10:**
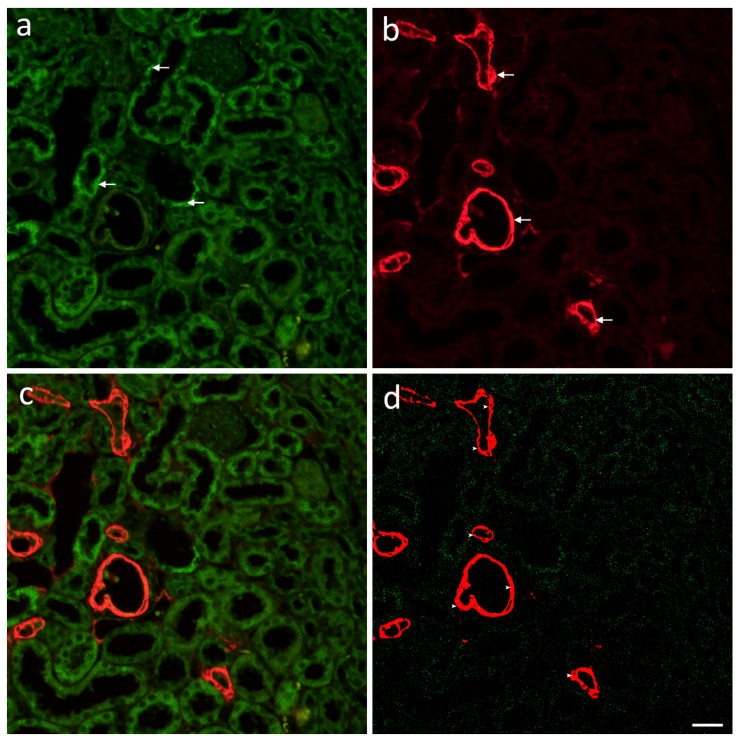
Immunofluorescence staining of postnatal *yotari* and wild type mouse kidneys with the Cx40 and aSMA markers. Positive signals (arrows) are shown on the green (**a**) and red (**b**) filter. Merged images (**c**) and isolated green and red thresholded signal (**d**) for better quantification of the section percentage area covered by positive signal and the co-localization with aSMA seen as yellow pixels (arrowheads). Scale bar is 20 μm, refers to all images.

To quantify connexin, pannexin and renin immunoexpression, ten non-overlapping representative visual fields of identical exposure time captured at an objective magnification of 40× were analyzed. Green staining, be it granular or diffuse cytoplasmatic, was interpreted as positive Cx40, Cx43, Cx37, Cx45, and Panx1 immunoexpression while red was considered positive for immunoexpression of alpha Smooth Muscle Actin (aSMA) and renin. ImageJ software (National Institutes of Health, Bethesda, MD, USA) was utilized for cell quantitative evaluation of immunoreactivity. Figures were prepared for analysis using subtraction of the median filter and color thresholding to measure the section percentage area covered by positive signal. Co-localization of Cxs and Panx1 with aSMA was calculated by dividing the area of overlap with aSMA area using Adobe Photoshop (Figure 10).

GraphPad Software (GraphPad Software, La Jolla, CA, USA) was utilized for statistical analyses with the probability level of *p* < 0.05 being regarded as statistically significant. A Two-way ANOVA test followed by the LSD post hoc test was used to compare immunoexpression in order to determine significant differences among groups.

### 4.5. Semi-Quantifcation of Cx Expression

The staining intensity of distinct kidney structures was semi-quantitatively analyzed into four groups: the absence of reactivity (−), mild reactivity (+), moderate reactivity (++), and strong reactivity (+++) (Table 2). For each investigated period, we captured at least twenty images per different kidney structure: proximal convoluted tubules (PCT), distal convoluted tubules (DCT), glomeruli and the medulla at 40× objective magnification. Any level of cytoplasmic or membrane staining with the used markers was regarded as positive. Three investigators analyzed the images independently.

## 5. Conclusions

In conclusion, substantial insights are emerging concerning the biology of Cxs and Panx, and their possible crucial role in the preservation of intercellular signaling within healthy kidney tissue as well as in the prevention of CKD.

Out of the results obtained in this study, we would like to highlight that renin granules were observed not only in the juxtaglomerular apparatus (JGA) but also inside the vascular walls of the glomeruli capillaries, in the proximal and distal convoluted tubuli as well as in the medulla resembling the distribution of renin cells in early embryonic and fetal life. The cause of this potential dedifferentiation might be the disturbance of Cx40 intercellular signaling that leads to a disbalance of blood pressure control through renin secretion and hypertension onset.

Further, we found a significant increase of Cx40 in the cortex of P4 *yotari* mice, which corresponded to normal renin expression. However, when this regulatory mechanism was exhausted, even normal expression of Cx40 at P14 *yotari* mice was not enough to prevent a high increase of renin expression at P14. Our conjecture is that the brightly fluorescing cells found in the glomeruli of P14 *yotari* mice are renin producing cells because Cx40 is known to be their most abundant connexin which displays strong expression. Overall, these results suggest an important role for connexins in the uncontrolled fashion of renin release in *yotari* mice.

Studies suggest that high blood pressure induces changes in the expression of vascular connexins in a region dependent fashion implying secondary changes in vascular function, thus implying possible hypertension could be the cause of the high expression of Cx43 and Cx45 in the vasculature of P14 *yotari* mice.

In our study, Cx37 and Cx40 displayed inverse appearance, with higher expression of Cx37 in comparison to Cx40 (in wt), while in *yotari* mice the expression of Cx40 was always higher than in wt, thus implying a regulatory role of Cxs in overcoming high renin expression in *yotari*. In addition, we also observed decreased Cx37 expression in the P14 *yotari* medulla that might contribute to hypertension reduction provoked by high renin expression.

Additionally, we suggest that loss of Dab1 might lead to the activation of c-Src tyrosine kinase, resulting in the upregulation of Cx43 and subsequent attenuation of renal fibrosis and electric abnormalities by activation of the cyclic adenosine monophosphate (cAMP) pathway. These findings implicate that the alteration of Cx expression in *yotari* mice is an early signal for the development of chronic kidney disease that ultimately leads to their premature death due to their possibly important role in cell responses to silenced Dab1 in *yotari* mice and their possible connection to the Dab1/Reelin signaling pathway. The remainder of our results might not be as pronounced, but they might show biological significance in future research.

Thus, our results point out the importance of Cxs in cell responses to silenced Dab1 in *yotari* mice and their possible connection to the Dab1/Reelin signaling pathway which might help establish certain connexopathies as the cause of premature death of this mouse line.

Our knowledge of their precise location in the kidney tissue combined with deeper characterization of these hemichannels proteins might lead to new therapeutic discoveries, but more detailed investigations are still to follow.

## Figures and Tables

**Table 1:** Staining intensity to specific antibodies in control and *yotari* mouse kidneys during the 4th and 14th postnatal day.

Postnatal Day	Part of Kidney	Cx37		Cx40		Cx43		Cx45		Panx1	
		WT	Y	WT	Y	WT	Y	WT	Y	WT	Y
4	G	+	+	++	+++	+	++	+	+	+	+
	PCT	++	++	+	+	+	+	+	++	+	+
	DCT	++	+++	++	++	+	+	++	+++	++	+++
	M	+	+++	+	++	+	+	++	+++	-	-
14	G	++	+	+	++	+	+	++	+	++	++/+++
	PCT	+++	+	+	+	+	+	+++	+++	++	++
	DCT	+++	++	++	++	++	++	+++	+++	++	+++
	M	+	+	+	++	+	+/++	++	+++	+	+

G—glomeruli; PCT—proximal convoluted tubules; DCT—distal convoluted tubules; M—medulla; WT—wild type; Y—*yotari* mice. Three pluses indicate strong reactivity; two pluses indicate moderate reactivity; one plus indicates mild reactivity; minus indicates no reactivity.

**Table 2:** Antibodies used for immunofluorescence.

Antibodies		Host	Dilution	Source
Primary	Anti-Cx37/GJA4 ab181701	Rabbit	1:500	Abcam (Cambridge, UK)
	Anti-Cx40/GJA5 ab213688	Rabbit	1:100	Abcam (Cambridge, UK)
	Anti-Cx43&GJA1 ab87645	Goat	1:200	Abcam (Cambridge, UK)
	Anti-Cx45/GJA7 ab135474 Anti-pannexin 1/PANX1	Rabbit Rabbit	1:100 1:300	Abcam (Cambridge, UK) Merck KGaA (Darmstadt, Germany)
	Smooth Muscle Actin (M0851) Renin ab134783 [7D3-E3]	Mouse Mouse	1:200 1:100	Dako (Glostrup, Denmark) Abcam (Cambridge, UK)
Secondary	Anti-Goat IgG, Alexa Fluor^®^ 488, ab150129	Donkey	1:400	Abcam (Cambridge, UK)
	Anti-Rabbit IgG, Alexa Fluor^®^ 488, 711-545-152	Donkey	1:400	Jackson Immuno Research Laboratories, Inc., (Baltimore, PA, USA)
	Anti-Mouse IgG, Rhodamine Red™-X, 715-295-151	Donkey	1:400	Jackson Immuno Research Laboratories, Inc., (Baltimore, PA, USA)

## Data Availability

The data presented in this study are available on request from the corresponding author.
